# Remaining Useful Life Estimation Using Deep Convolutional Generative Adversarial Networks Based on an Autoencoder Scheme

**DOI:** 10.1155/2020/9601389

**Published:** 2020-08-01

**Authors:** Guisheng Hou, Shuo Xu, Nan Zhou, Lei Yang, Quanhao Fu

**Affiliations:** College of Economics and Management, Shandong University of Science and Technology, Qingdao 266590, China

## Abstract

Accurate predictions of remaining useful life (RUL) of important components play a crucial role in system reliability, which is the basis of prognostics and health management (PHM). This paper proposed an integrated deep learning approach for RUL prediction of a turbofan engine by integrating an autoencoder (AE) with a deep convolutional generative adversarial network (DCGAN). In the pretraining stage, the reconstructed data of the AE not only participate in its error reconstruction but also take part in the DCGAN parameter training as the generated data of the DCGAN. Through double-error reconstructions, the capability of feature extraction is enhanced, and high-level abstract information is obtained. In the fine-tuning stage, a long short-term memory (LSTM) network is used to extract the sequential information from the features to predict the RUL. The effectiveness of the proposed scheme is verified on the NASA commercial modular aero-propulsion system simulation (C-MAPSS) dataset. The superiority of the proposed method is demonstrated via excellent prediction performance and comparisons with other existing state-of-the-art prognostics. The results of this study suggest that the proposed data-driven prognostic method offers a new and promising prediction approach and an efficient feature extraction scheme.

## 1. Introduction

As the demand for reliability and efficiency in maintenance technical areas is increasing, prognostic and health management (PHM) has received significant attention. PHM is not only able to decrease the rate of accidents occurring and prolong the lives of devices by replacing old or broken components with new ones earlier but also able to avoid wasting resources by canceling unnecessary maintenance activities [1]. The most common task of PHM is to predict the remaining useful life (RUL) of important components and systems in different environments. RUL is the length of time from the current time to the time that the components or systems break down. If the RUL of the components or systems can be predicted accurately, appropriate maintenance actions can be scheduled proactively to avoid catastrophic failures and minimize the economic losses of systems [2]. This paper proposes a novel deep learning scheme for improving RUL estimation. The DCGAN-based AE model is trained in an unsupervised way to convert the multisensor (high-dimensional) readings collected from historical run-to-failure instances (i.e., multiple units of the same system) to low-dimensional features, which include important degradation information of the original data. Then, a long short-term memory (LSTM) network is used to capture the sequential information from the extracted representations to predict the RUL.

Generally, the methods of dealing with RUL prediction problems can be categorized into model-based approaches, sensor-based data-driven approaches, and hybrid approaches. For model-based methods, it is difficult to model extremely complicated systems, such as aircraft systems. Moreover, model-based methods require a large amount of prior knowledge and expertise, which limit the effectiveness of these methods. For sensor-based data-driven approaches, the availability of sufficient information is the necessary condition to maximize their powerful processing capability. Fortunately, it is quite common in the current era for a large number of sensors to be installed to monitor the operational behaviors of a system. These data records are historical observations that can be exploited as useful information. Hybrid methods usually combine the two aforementioned methods. However, it still remains very challenging to utilize the advantages and avoid the disadvantages of both approaches. Therefore, the method adopted in this paper is a data-driven approach.

Currently, most data collected in real-life PHM applications are high-dimensional. Due to their complicated environment, monitoring data are subjected to several operating conditions and fault modes, increasing the inherent degradation complexity and the difficulty of directly discovering clear trends in the input data for the prognostic algorithm. To cope with this issue, feature extraction is a necessary procedure to capture useful information from high-dimensional data efficiently [3]. Among the deep learning architectures, convolution neural networks (CNNs), which are specifically designed for variable and complex signals, are the basis of the feature extraction in this study.

The CNN was first proposed by LeCun et al. for image processing [4]. The ability of the CNN to maintain data information regardless of scale, shift, and distortion invariance is presented. CNNs not only performed excellently on computer vision tasks [5], such as object recognition [6] and face recognition [7], but also can be applied to multichannel sequential sensor data. The deep CNN architecture has been proven to be effective for extracting abstract information by Li et al. [8]. Five convolution layers are stacked to learn the high-level representations from different sensor measurements, and a good prognostic performance is achieved. Since the deep CNN structure has shown a remarkable feature extraction ability [9], it is chosen as the basic tool to capture high-level abstract features in this study.

An autoencoder neural network [10] is another representation extraction method, whose architecture consists of an input layer, a hidden layer, and an output layer. The AE is a neural network based on an unsupervised learning algorithm that can convert input data into a lower-dimensional representation. Malhotra et al. proposed an LSTM-based autoencoder scheme to obtain an unsupervised health index (HI), which is used to predict the RUL via comparisons. The experimental results showed that this method performs better than other methods [11]. Ren et al. utilized the AE to construct a multidimensional feature extraction model that represents battery health degradation. Next, the higher RUL prediction accuracy of the lithium-ion battery is obtained by the deep neural network [12].

To extract more degradation-related features, a deep CNN is embedded in the AE as the basic neural network architecture. It is difficult for the AE to extract deeper abstract information in a single-error reconstruction process. To strengthen the feature extraction capability of the AE, it is used as a generator to participate in the training process of the DCGAN, and its parameters are trained and optimized again. Through double-error reconstructions, the high-level abstract representation is captured and revealed the underlying correlations and causalities in the collected sensor data. Generative adversarial networks (GANs) were proposed by Goodfellow et al. [13] in 2004 as a new framework for estimating generative models via an adversarial process. GANs have achieved impressive results in image generation [14] and image editing [15]. The DCGAN [16] has a more stable architecture than a GAN by applying some constraints on GANs. While the DCGAN has shown a strong data generation ability, very limited research can be found on its applications to machinery RUL prediction problems to extract high-level abstract features.

As one of the most complex systems, aircraft systems have always been a focus of health monitoring. The engine is one of the most important components for determining the health and life of an aircraft. Hence, there is always a pressing need to develop new approaches to better evaluate engine performance degradation and estimate its RUL. Our work meets this need by proposing a new deep learning model.

In this paper, the time window approach is employed to prepare samples to conduct better feature extraction via the DCGAN based on an AE pretraining model. Raw sensor measurements with normalization are directly used as inputs to the proposed model, and no prior expertise on prognostics or signal processing is required, which facilitates the industrial application of the proposed method. After high-level abstract features are extracted by the pretraining model, the associated RUL is estimated based on the learned representations via an LSTM. Through a double-nested error regression, more degradation-related features can be exploited in the pretraining stage, which is helpful for the whole algorithm to better understand the underlying degradation phenomena.

In view of the effectiveness of our proposed pretraining model, the proposed method is expected to obtain a higher prognostic accuracy than other deep learning methods. A comprehensive analysis of the proposed approach and comparisons with existing methods are presented in this study. The results are verified on four different simulated turbofan engine degradation datasets from the publicly available commercial modular aero-propulsion system simulation (C-MAPSS) dataset produced and provided by NASA [17]. This study's main contributions are as follows:

1. This paper innovatively integrates an AE and a DCGAN as a pretraining model, which greatly enhances the ability of feature extraction. Through double-error reconstruction, the generated data are closer to the original data so that the intermediate features extracted by the encoder contain more useful information of the original data. Although the simple LSTM and fully connected neural networks (FNNs) are chosen as the fine-tuning stage, better prediction performance is still achieved, which proves the effectiveness of our proposed pretraining model. It is suitable not only for engine datasets but also for other datasets as a feature extraction framework. This work could provide a new perspective to study unsupervised feature representation methods.
2. The proposed new algorithm achieves the RUL prediction performance compared to other comparative algorithms with several operating conditions and fault modes. The proposed algorithm is appropriate for RUL prediction. Higher prediction accuracy allows an enterprise to arrange maintenance activities in advance, which improves the reliability of the system and the economy of the enterprise.

The remainder of this paper is structured as follows. Related work on RUL prediction is introduced in Section 2. The proposed deep learning structure and the necessary constituent components are described in Section 3. In Section 4, the experimental results and evaluations are compared with other popular methods to show the effectiveness and superiority of the proposed architecture. Finally, conclusion and discussion are provided to close the paper.

## 2. Related Work in the C-MAPSS Dataset

The C-MAPSS dataset has been extensively used to evaluate the effectiveness of deep learning algorithms for RUL estimation. This section reviews the most recent studies conducted using the C-MAPSS dataset. Then, the proposed method is briefly introduced, which is compared with these studies in a later section.

### 2.1. Related Work

In most PHM applications, sensor data are easy to obtain for intelligent machine health monitoring. Sequential data are the common format of input data. In deep learning, the recurrent neural network (RNN) [18] is an important technique to deal with sequential data, and it has been widely used to generate sequences in domains including speech recognition and text translation. However, the RNN lacks the capability to learn long-term dependencies because of the vanishing and exploding gradient issue. Its variant—LSTM [19]—which contains a memory cell that regulates the information flow in and out of the cell—can deal with the problem of the RNN well. Zheng et al. [20] proposed a model that combines multiple layers of LSTM cells with standard feedforward layers to reveal hidden patterns within the sensor and operational data. The model showed better performance than the multilayer perceptron (MLP), support vector regression (SVR), and relevance vector regression (RVR). Another LSTM approach in which dynamic technology was used to extract new features from raw sensor data before the training procedure was proposed by Wu et al. [21]. This method provides the RUL estimation using a vanilla LSTM and obtains a higher prediction accuracy than the standard RNN and gated recurrent units (GRU) LSTM under the same number of hidden neurons in a single layer.

To deal with sequential information more effectively, a CNN can be used to extract abstract features before LSTM layers. Although CNNs have performed excellently on computer vision tasks, such as object recognition [22] and face recognition [7], CNNs can also be applied to multichannel sequential sensor data. Babu et al. [23] proposed a novel model that includes two layers with convolution and average-pooling modules and an FNN to perform RUL predictions. Another CNN approach in which a time window approach was employed for sample preparation was provided by Li et al. [8]. Five convolution layers are stacked in the network, and the final feature map is the same size as the input because of zero padding. The higher prediction accuracy is demonstrated by comparisons with the RNN, LSTM, and so on.

It is easier to capture the latent representation of the original data in the form of a pretrained model. Thus, a semisupervised learning method is suitable for RUL estimation. Ellefsen and colleagues [24] introduced a semisupervised architecture to predict the RUL in which a restricted Boltzmann machine (RBM) [25] serves as the initial pretraining stage to extract degradation information from unlabeled sensor data. To tune the hyperparameters in the training stage, a genetic algorithm (GA) approach is applied, which achieves a higher prediction accuracy than the popular supervised learning. Moreover, Yoon et al. [26] described a semilearning approach that uses nonlinear embedding based on the variational autoencoder (VAE) model. With this approach, they achieved good prediction performance, even when the available label information was highly limited.

To improve the efficiency of solving problems, different deep learning tools are combined. Yu et al. obtained one-dimensional HI values from sensor data via the bidirectional RNN-based autoencoder, which represents the degradation patterns of the units of the system. Then, they used the similarity-based curve matching technique to estimate the RUL [27]. Zhang et al. combined the deep belief network (DBN) training technique with a multiobjective evolutionary algorithm to evolve multiple DBNs with varying accuracies and diversities. Then, an ensemble model is created by evolved DBNs to predict the RUL [28]. The recent studies are summarized in Table 1.

### 2.2. Proposed Methodology

**Figure 1:**
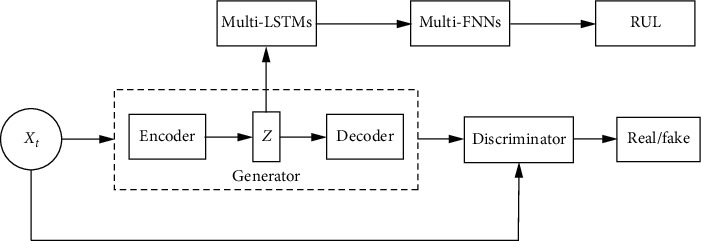
The architecture of semisupervised learning.

As stated previously, there is great potential to improve the RUL estimation accuracy by extracting intermediate representations. This paper proposes a new double-nested pretraining model that enhances the quality of the extracted intermediate representation. As shown in Figure 1, the pretraining model is composed of a DCGAN based on an AE scheme in which the AE is embedded in the DCGAN as the generator. The key to the success of a GAN is the concept of adversarial loss, which forces the generated data to be, in principle, indistinguishable from real data. The proposed pretraining model combines the adversarial loss of the DCGAN with the error reconstruction of the AE to learn the mapping such that the generated data are hard to distinguish from raw data. Through double-error training, the generated data are closer to the original data, which ensures that the intermediate features extracted by the encoder contain important original information. Next, to highlight the efficiency of the extracted abstract features, the simplest two-layer LSTM is used to further capture the temporal information of the features instead of the bidirectional LSTM in the supervised learning stage. Finally, the following fully connection layers predict the target RUL.

**Figure 2:**
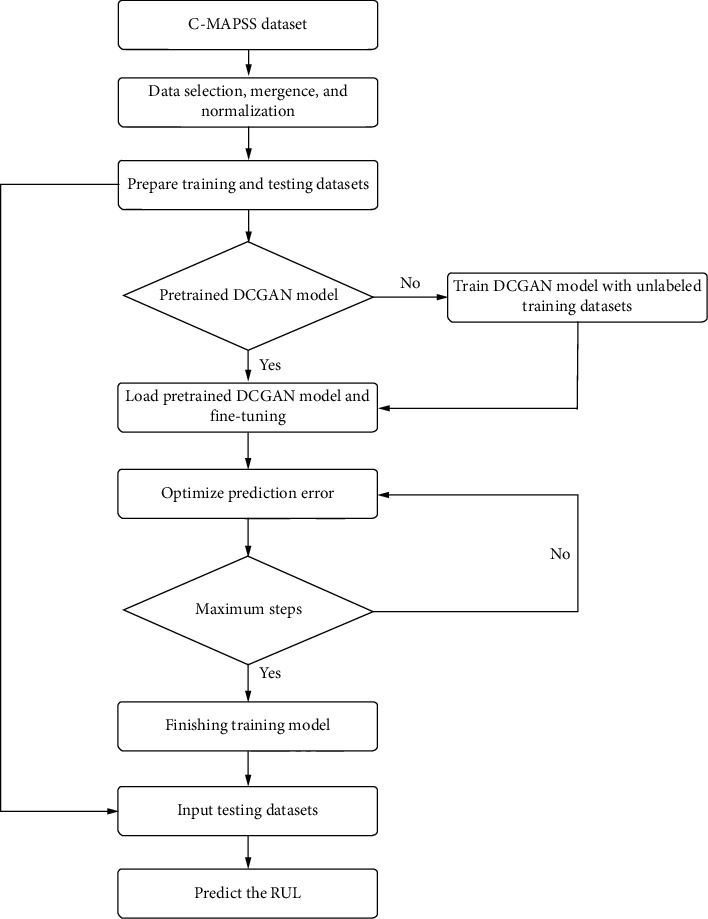
Flowchart of the proposed architecture for prognostics.

The specific flowchart of the proposed method is illustrated in Figure 2. Before inputting the engine data into the model, the data are first preprocessed, including data selection, fusion, and standardization. The specific content is introduced in detail in the experimental study section. The training data and test data are prepared with the sliding window method. Then, the pretraining model is trained first to obtain latent features. The target RUL is output via supervised learning in the fine-tuning stage. After training is completed, the test data are input into the model to predict the RUL and verify the prediction performance.

The monitoring data of complex systems, such as engine data, have the characteristics of high dimensions. Due to the influence of various operational conditions and fault modes, it is difficult for the model to directly capture the hidden degradation trend in data, which decreased the prediction accuracy of the model. Therefore, it is necessary to perform high-level feature extraction on the data. The pretraining model proposed in this paper combines a DCGAN and an AE to form a double-nested feature extraction structure, which greatly improves the quality of the extracted features and thus improves the prediction accuracy of the model.

Aircraft engines are typical complex systems. C-MAPSS is the benchmark dataset for detecting the prediction performance of the RUL of aircraft engines. It includes software simulated data of the failures and degradation of large commercial turbofan engines under different operating conditions. Our comprehensive experiments and comparisons with recently proposed RUL estimation algorithms developed based on this dataset show the superiority. The details of the comparison are shown in a later section.

## 3. Constituent Components and the Proposed Scheme

This section introduces the necessary components of the proposed model architecture. First, the main deep learning tools are introduced, including the CNN, autoencoder, LSTM, and DCGAN. Next, the architecture of the proposed model is elaborated.

### 3.1. Convolutional Neural Network

The CNN was first proposed by LeCun for image processing, and the network has three characteristics, i.e., local receptive fields, tied weights, and spatial subsampling [29]. CNNs have achieved significant success in the field of computer vision [30], where input data are 2-dimensional (2D). A CNN is also useful when input data are 1-dimensional (1D) [31, 32], which is why CNNs are utilized in natural language processing and speech recognition [33, 34]. A classical convolutional module consists of convolutional layers and pooling layers in which multiple filters extract spatial features, and pooling operations choose the most significant information. Actually, the pooling layers are increasingly replaced by strided and fractionally strided convolutions to maintain more useful information from the feature maps. Johnson et al. and colleagues [35] trained a feedforward transformation network with a perceptual loss function that improved the style-transfer and single-image super-resolution performances.

In this study, the input data are prepared in a 2D format, where one dimension is the number of sensors, and the other is the time sequence of each feature. Despite the fact that the sources of the collected features are different sensors, the relationship between the spatially neighboring features in the data sample is not remarkable. Thus, the convolution filters in the proposed model are considered to be 1-dimensional (1D) in the first four layers. In the following, the 1D CNN is briefly introduced.

First, the input sequential data are assumed to be *x* = [*x*_1_,…, *x*_*T*_], where *T* is the length of the sequence. The convolution operation in the convolutional layer is determined by using multiplication between a kernel *w* ∈ *R*^*D*×1^, where *D* is the kernel size; the concatenation vector representation *x*_*i*:*i*+*D*−1_^*t*^ is given by the following:

(1) $$\begin{array}{c} x_{i:i+D-1}^{t}=x_{i}^{t}⊕x_{i+1}^{t}⊕\cdots ⊕x_{i+D-1}^{t}, \end{array}$$

where *x*_*i*:*i*+*D*−1_^*t*^ represents a window of length *D* that starts from the *i*th point and ⊕ concatenates each data sample into a vector. Therefore, the convolution operation is defined as follows:

(2) $$\begin{array}{c} z_{l}^{t}=\varphi \left(w^{T}x_{i:i+D-1}^{t}+b\right), \end{array}$$

where superscript *T* denotes the transpose operator and {*b*, *φ*} are, respectively, the bias and nonlinear activation functions. By denoting *z*_*l*_^*t*^ to represent the learned feature of kernel *w* on the subsequence *x*_*i*:*i*+*D*−1_^*t*^ and by sliding the filtering window from the first point to the last point in the sampled data, the feature map *z*_*j*_^*t*^ of the *j*th kernel can be captured and is expressed as follows:

(3) $$\begin{array}{c} z_{j}^{t}={\left[z_{1}^{t},z_{2}^{t},\ldots ,z_{l-D+1}^{t}\right]}^{T}. \end{array}$$

### 3.2. Autoencoder

An autoencoder (AE) is a typical unsupervised learning method aimed to extract abstract representations from raw data, and it includes three essential parts: an encoder, representations, and a decoder. The input data *X* are processed by an encoder to obtain the hidden representation *z* that contains abstract information:

(4) $$\begin{array}{c} z=\varphi \left(X\right), \end{array}$$

where *φ* is a nonlinear activation function. Then, the decoder maps representation *z* to the outputs, which is similar to the input data:

(5) $$\begin{array}{c} H=\varphi^{\prime }\left(z\right), \end{array}$$

where *φ*′ is a nonlinear activation function. By minimizing the reconstruction error between the input data *X* and the output *H*, the representation *z* can be regarded as the high-level abstract features of the input data that contain the important original information. The representations extracted by the AE have been utilized to process RUL prediction problems, such as in [36, 37].

### 3.3. Long Short-Term Memory

An LSTM is a variant of an RNN that aims to address sequential data. LSTMs have achieved great success on speech recognition and machine translation [38]. By controlling the information flow via an input gate, forget gate, and output gate, LSTMs overcome the shortcoming of the RNN, which is not able to deal with long-term time dependency.

The core structures of an LSTM cell are three nonlinear gating units. Forget gates control the forget rate of the last cell information and are denoted as *f*_*t*_:

(6) $$\begin{array}{c} f_{t}=\sigma \left(W_{f}\cdot \left[h_{t-1},x_{t}\right]+b_{f}\right). \end{array}$$

Input gates (*i*_*t*_) decide how much new information can be added:

(7) $$\begin{array}{c} i_{t}=\sigma \left(W_{i}\cdot \left[h_{t-1},x_{t}\right]+b_{i}\right). \end{array}$$

Output gates (*o*_*t*_) are responsible for the output proportion of cell memory:

(8) $$\begin{array}{c} o_{t}=\sigma \left(W_{o}\cdot \left[h_{t-1},x_{t}\right]+b_{o}\right), \end{array}$$

where *σ* is the sigmoid gate activation function that obtains a scaled value between 0 and 1; *W*_*f*_, *W*_*i*_, and *W*_*o*_ are the input weights; *b*_*f*_, *b*_*i*_, and *b*_*o*_ are the bias weights; and · is the matrix multiplication of two vectors. The new candidate state values, ${\tilde{C}}_{t}$, are created by the tan*h* layer:

(9) $$\begin{array}{c} {\tilde{C}}_{t}=\tan h\left(W_{c}\cdot \left[h_{t-1},x_{t}\right]+b_{c}\right). \end{array}$$

The candidate state values ${\tilde{C}}_{t}$are combined with the previous cell state *C*_*t*−1_ to generate the new cell state *C*_*t*_:

(10) $$\begin{array}{c} C_{t}=f_{t}*C_{t-1}+i_{t}*{\tilde{C}}_{t}, \end{array}$$

where *∗* denotes elementwise multiplication of two vectors. The forget gate *f*_*t*_ keeps a portion of the historical information. Then, the input gate *i*_*t*_ determines which new information in ${\tilde{C}}_{t}$ will be updated and saved in *C*_*t*_.

The output gate, *o*_*t*_, decides how much the memory cell *C*_*t*_ will output. *C*_*t*_ ranges from −1 to 1.

(11) $$\begin{array}{c} h_{t}=o_{t}*\tan h\left(C_{t}\right). \end{array}$$

Through these steps, the LSTM cell is updated in every step.

## 4. Generative Adversarial Network and Deep Convolutional Generative Adversarial Network

The core idea of the GAN is the adversarial loss formulated by the generator model *G* and the discriminator model *D*. The generator *G* is used to generate an image from random noise to try to cover the real-data distribution. In addition, *D* tries to determine whether the image comes from real datasets or generated by *G*.

*G* and *D* formulate the two-player mini-max game in which *G* tries to capture the distribution of real data *x* to fool discriminator *D*, and *D* is trained to detect whether the generator's output is fake. To achieve the goal mentioned above, *D* is trained to maximize log{*D*(*x*)}, and the parameters of *G* are adjusted to minimize log(1 − *D*(*G*(*z*))). The total adversarial loss can be described as follows:

(12) $$\begin{array}{c} \underset{G}{\min}\underset{D}{\max}V\left(D,G\right)=E_{x∼p\;\mathrm{data}\;\left(x\right)}\left[\log D\left(x\right)\right]+E_{z∼p_{z}\left(z\right)}\left[\log\left(1-D\left(G\left(z\right)\right)\right)\right]. \end{array}$$

DCGANs have a more stable architecture than GANs via five improvements. First, the pooling layers are replaced with strided convolutions (discriminator) and fractional-strided convolutions (generator). Second, the batch normalization method is used in both the generator and the discriminator [39]. Third, the fully connected hidden layers for deeper architectures are removed. Fourth, rectified linear unit (ReLU) activation is used in the generator for all layers except for the output, which uses tan*h* [25]. Finally, LeakyReLU activation is utilized in the discriminator for all layers.

### 4.1. The Proposed Model Architecture

Temporal sequence data provide more information in comparison to a multivariate data point sampled at a single time step. In the proposed architecture, therefore, a sliding window strategy is adopted to use multivariate temporal information efficiently. The input of the proposed model is a 2D matrix *x*_*i*_ containing *x*_*tw*_ (the size of the sliding window) with *x*_*f*_ (the number of the selected features). Different values of *x*_*f*_ are considered for different subdatasets of the C-MAPSS dataset. For subsets FD001 and FD003, the value of *x*_*f*_ is set equal to 16, and the value of *x*_*f*_ for subsets FD002 and FD004 is 24. *x*_*tw*_ is equal to 32 for all subsets. Moreover, the step size of the sliding window is chosen to be 1. The segmented multivariate time series matrix (*x*_*f*_ × *x*_*tw*_) is fed into the proposed model.

The proposed architecture structure is shown in Figure 1. To learn the abstract representations of input data *x*, the DCGAN model is pretrained using unsupervised learning before the prediction of the RUL. The generator of the DCGAN is composed of an AE architecture in which the encoder is used to generate hidden representations of input data, and the decoder reconstructs input data to fool the discriminator. The generation error and reconstruction error are utilized together for feature extraction. Through the dual optimization of the DCGAN and AE, the representations extracted by the encoder can capture more useful information than only one. Then, the LSTM and FNN are combined to output the target RUL in the fine-tuning stage.

**Figure 3:**
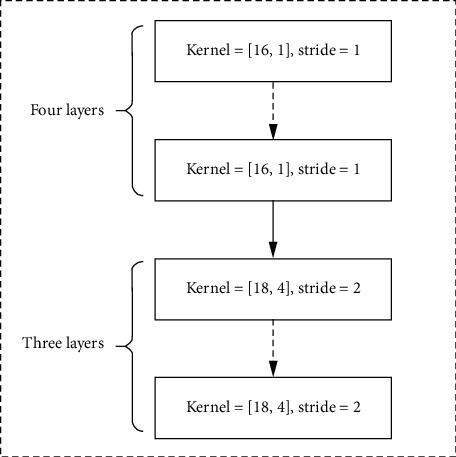
The architecture of the encoder.

Considering that the input data are collected from different sensors, four convolutional layers with 1-dimensional convolution filters and zero paddings are stacked to extract the degradation information inside every sensor observation in the generator. The first four CNN layers consist of 10 filters (16 × 1). The relationship between the spatially neighboring features in the data sample is captured by three stride-2 convolutions with 18 × 4 filters. The ReLU function is used for the convolution layers. Seven total convolutional layers constitute the encoder of the AE model. The structure is shown in Figure 3.

To maintain the same size of the raw data *x* for the output data, three (1/2)-stride convolutions are stacked in the decoder. All convolutional layers in the AE employ the ReLU as the activation function, except that the last convolutional layer of the decoder uses the tan*h* function. Moreover, the discriminator has the same configurations as the encoder except for the activation function. The tan*h* function is used for the final convolution layer. The discriminator *D* is used to distinguish the input data *x* from the generated data. When *D* fails to discriminate the generated data, the generator is successful at capturing the real distribution of the real data, which means that the extracted representation *z* consists of the important information of the real data. It is meaningful to predict the RUL.

Note that the dropout technique is used in the first convolutional layer in the decoder and the first FNN to relieve overfitting [40]. Both the generator and the discriminator use convolution-BatchNorm-ReLu modules except for the output layers. Moreover, the Xavier normal initializer is utilized for the weight initializations [41]. The RMSprop algorithm [42] is employed for optimizing the adversarial loss in the unsupervised pretrained process.

In the supervised stage, two LSTM layers are used to reveal the hidden sequential features of the representation *z* generated by the pretraining stage. Each of the two layers is defined by a 64-cell structure. Repeating cells within each LSTM layer have the same structure and parameter values. Then, two FNNs are stacked to map all the extracted features to the RUL. To improve the prognostic accuracy, a fine-tuning process algorithm is applied via backpropagation (BP) [43], where the parameters of the supervised learning model are updated to minimize the prediction error. The Adam algorithm is employed for optimization [44]. The entire process of model training is shown in Algorithm 1.

## 5. Experimental Study

In the following experimental study, the performance of the proposed framework is evaluated. First, we introduce the C-MAPSS dataset, which has been adopted by many studies. Then, the details of the experimental setup are elaborated. Finally, comparison results are shown and discussed. All the experiments are run on an Intel(R) Core(TM) i7-8550U with 8 GB of RAM and the Microsoft Windows 10 operating system. The programming languages for the deep learning are “TensorFlow” version 1.13.1 and Python 3.5 [45].

### 5.1. C-MAPSS Dataset

C-MAPSS is a dataset that simulates the effects of faults and deterioration under different operating conditions in the five main rotating components (fan, low-pressure compressor, high-pressure compressor, high-pressure turbine, and low-pressure turbine) found in a large commercial turbofan engine. The C-MAPSS dataset consists of 4 subsets, which are divided into training datasets and test datasets. Each subset includes 26 columns: the number of engines, operational cycles, three operational sensor settings, and 21 sensor measurements that give 21 types of measurements from 21 sensors. A description of the sensed engine variables can be found in Table 2. The three operating mode indicators are altitude, Mach number, and throttle resolver angle, which determine different flight conditions of an aero-engine.

In addition, different subsets have different numbers of engines whose operational cycles vary. Each engine starts with different degrees of initial wear and manufacturing variation that are unknown and considered to be healthy. As the operating time increases, the engines start to degrade at some point. The degradation in the training datasets grows in magnitude until a failure occurs, while the degradation in the test datasets ends sometime prior to the occurrence of a failure, which is the RUL. The purpose of the proposed algorithm is to predict the RULs of the test datasets. To access the prediction accuracy, the true RUL targets of the test datasets are provided.

The basic information of the datasets is given in Table 3. Specifically, FD001 represents a situation in which a fleet of engines suffered a high-pressure compressor failure with a single operating condition, FD002 represents a situation in which the engines suffered a high-pressure compressor failure with six operating conditions, FD003 is the situation in which a fleet of engines suffered high-pressure compressor and fan degradations with a single operating condition, and FD004 is the situation in which a batch of engines suffered high-pressure compressor and fan degradations with six operating conditions (see [46] for more details).

In the training process, all the available engine measurements are used as the training samples, and the corresponding RUL labels obtained from a piecewise linear degradation model are regarded as the targets [47]. During the test stage, the performance at the last time step for each engine is generally used as the testing sample. The actual RUL of the testing samples is provided to calculate the prognostic accuracy.

### 5.2. Feature Selection

Sensors 1, 5, 6, 10, 16, 18, and 19 in subsets FD001 and FD003 exhibit constant sensor measurements throughout the engine's lifetime, which are not important for RUL estimation. In addition, subsets FD001 and FD003 are subject to a single operating condition. Hence, the three operational settings are excluded. Accordingly, sensors 2, 3, 4, 7, 8, 9, 11, 12, 13, 14, 15, 17, 20, and 21 are used as the input features for subsets FD001 and FD003. However, to make the output data generated by the decoder maintain the same size as the input data, one of the constant sensor measurements needs to be used. Any one of six constant sensors is sufficient, and thus, sensor 19 is chosen is this paper.

The three operational settings cannot be excluded due to the six operating conditions in subsets FD002 and FD004. Only sensor 1 is dropped from the constant measurements to recover the input data in the generator. In fact, any one of the constant sensor measurements is sufficient.

### 5.3. Merging of Training Datasets

It is obvious that FD001, FD002, and FD003 are particular cases of FD004 [48]. There is an increasing order of complexity from FD001 to FD004. To obtain better results and demonstrate the generalization capacity, simple datasets can be merged into complex ones to increase the sizes of the datasets when the representation is abstracted in the unsupervised training stage. However, the test datasets are not merged because the goal of this paper is to demonstrate the superiority of the proposed method over other methods.

No datasets are merged with dataset FD001 since it is useful to test the generalization capacity of the model in the simplest case. Moreover, the algorithm needs to generalize more complex cases, such as FD002 and FD004, in which all the subdatasets are used for their unsupervised training. For dataset FD003, FD001 is merged with it for the sake of measuring the capability of dealing with multiple fault modes [49].

### 5.4. Data Normalization

For each of the 4 subdatasets in C-MAPSS, the collected measurement data from each sensor are normalized to be within the range of [−1, 1] using the min-max normalization method:

(13) $$\begin{array}{c} x_{\text{norm}}^{i,j}=\frac{2\left(x^{i,j}-x_{\min}^{j}\right)}{x_{\max}^{j}-x_{\min}^{j}}-1,\;\forall i,j, \end{array}$$

where *x*^*i*,*j*^ denotes the original *i*-th data point of the *j*-th sensor and *x*_norm_^*i*,*j*^ is the normalized value of *x*^*i*,*j*^. *x*_max_^*j*^ and *x*_min_^*j*^ denote the maximum and minimum values of the original measurement data from the *j*-th sensor, respectively.

### 5.5. Label Training Datasets

**Figure 4:**
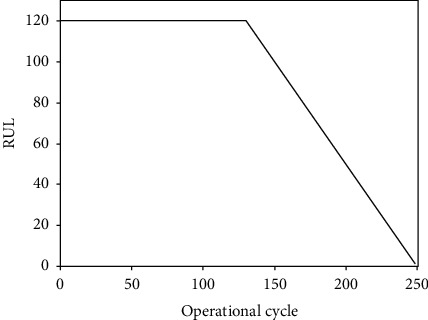
Illustration of the piecewise linear degradation function.

True RUL labels are not provided in the training sets; they are only provided at the last time step for each engine in the test sets. To construct labels for every time step for each engine in the training sets, a piecewise linear degradation model has been validated to be suitable and effective to label training datasets [50]. In general, an engine works normally in the early stages and degrades linearly afterward and is assumed to have a constant RUL label in the initial period (Figure 4).

**Figure 5:**
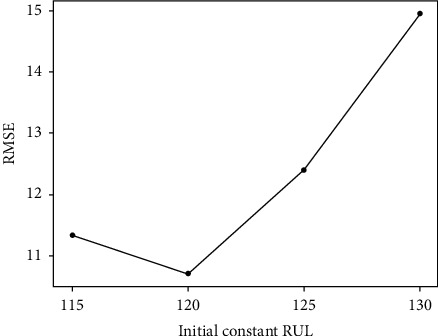
Prediction performance of different RUL values on FD001.

This piecewise linear RUL target function is the most common approach in previous studies. Based on previous studies, the choice of the initial constant RUL is mainly divided into four types, namely, 115, 120, 125, and 130. The experimental results of the four parameter settings are shown in Figure 5. It is clear that the prediction performance is the best when RUL is set to 120.

### 5.6. Performance Metrics

**Figure 6:**
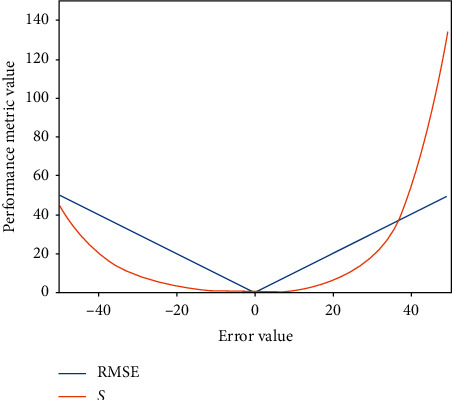
Difference between RMSE and *S* with respect to different errors.

For the sake of comparability with other algorithms, the same performance metrics are used to evaluate the prediction accuracy. The formulas for the scoring function (*S*) and root mean square error (RMSE) are provided in Saxena et al. [46]:

(14) $$\begin{array}{c} S=\left\{\begin{array}{cc} \sum_{i=1}^{n}e^{\left(-\left(d_{i}/13\right)\right)}, & \mathrm{for}\;d_{i}<0,\\ \sum_{i=1}^{n}e^{\left(-\left(d_{i}/10\right)\right)}, & \mathrm{for}\;d_{i}\geq 0, \end{array}\right.\\ \mathrm{RMSE}\;=\sqrt{\frac{1}{n}\sum_{i=1}^{n}d_{i}^{2}}, \end{array}$$

where *n* is the total number of true RUL targets in the respective test set and *d*_*i*_=RUL_predicted_ − RUL_true_. The RMSE gives an equal penalty to early and late predictions. The scoring function penalizes late predictions more than early predictions because late predictions usually lead to more severe consequences in many fields, such as the aerospace industry. In comparison, early predictions pose less risk, but they lead to greater costs. Nevertheless, the main objective is to achieve the smallest value possible for both *S* and RMSE, that is, when the error value *d*_*i*_=0.  Figure 6 shows the details.

### 5.7. Configuration of the Proposed Architecture

**Figure 7:**
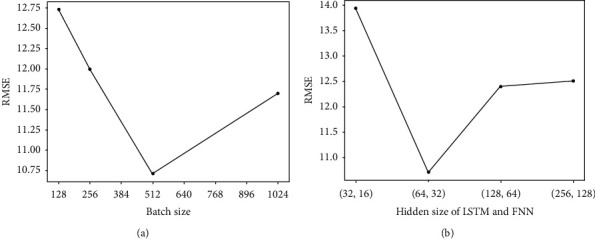
Prediction performances of different hyperparameters in FD001. (a) Prediction accuracy of different batch sizes in FD001. (b) Prediction accuracy of different hidden sizes in FD001.

First, the C-MAPSS subdatasets are preprocessed as mentioned above. The normalized data are sent to the initial DCGAN based on the AE pretrained model to extract high-level abstract representations, which are used to predict the RUL. Due to the large amount of data, minibatches are used to train the model. The value of the hyperparameter batch size has an impact on the prediction performance. We choose common values for the experiment (values: 128, 256, 512, and 1024). The results are shown in Figure 7(a). It is obvious that the best performance is obtained when 512 is selected. Three thousand batches are generated to train the initial pretrained model.

Then, the extracted features are utilized by two LSTM layers and two FNN layers to predict the RUL. To achieve better prediction performance, four commonly used hidden node numbers are evaluated ((32, 16), (64, 32), (128, 64), and (256, 128)). When the hidden nodes of the LSTM and FNN are 64 and 32, respectively, the highest prediction accuracy is achieved (see Figure 7(b)). Since the last layer of the FNN outputs the target RUL, the hidden size is set to 1. Backpropagation learning is used to fine-tune the weights in the network. The Adam optimization algorithm is used with minibatches for the updates. For the sake of stable convergence, the learning rate is 0.0002.

The hyperparameter *p* for the dropout technique is used to randomly drop units during training. In this way, dropout approximately combines an exponential number of different architectures, which enhances the feature extraction ability and alleviates overfitting. A typical value for *p* used in the literature is 0.5, which is the value chosen in this paper. As Patterson and Gibson [51] recommended, to preserve the important extracted features, dropout is disabled in the first layer and output layer. In the proposed method, dropout is used in the first transposed convolutional layer of the decoder, the two LSTM layers, and the first fully connected layer of the supervised architecture. The parameters of the supervised architecture are presented (see Table 4).

In the training procedure, each complete training subset is split into a training set and a cross-validation set. Fifteen percent of the total time windows in the training subsets are randomly selected for cross-validation. The remaining 85% of the total data are designated as the training sets. After the pretraining stage, the testing data samples are fed into the trained network for the RUL prognostics. Finally, the target RUL and prediction accuracy can be obtained.

## 6. Experimental Results and Discussion

In this section, the performance of the proposed deep learning approach is evaluated. First, the prediction results of four subsets are analyzed. Then, a comparison is conducted with other state-of-the-art methods to show the superiority of the proposed approach.

### 6.1. RUL Estimation Results

**Figure 8:**
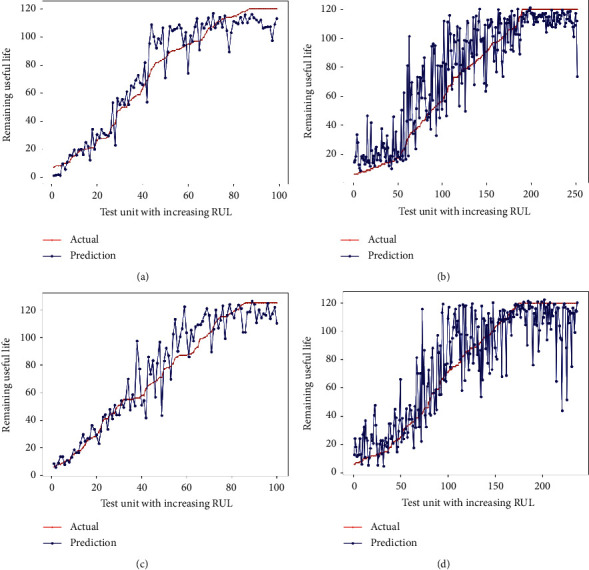
Prediction for the last recorded data point of different testing engine units in FD001–FD004: (a) prediction for the 100 testing engine units in FD001; (b) prediction for the 256 testing engine units in FD002; (c) prediction for the 100 testing engine units in FD003; (d) prediction for the 248 testing engine units in FD004.

The RUL prediction results over the four datasets (i.e., FD001–FD004) are presented in Figures 8(b)–8(d). To better visualize the results, in the figures, the testing engines are sorted in the ascending order (from small to large). Figures 8(b)–8(d) show the prediction results associated with the last recorded data point over the four datasets. It is worth mentioning that the number of test cases in each dataset is different, ranging from 100 testing engines in FD001 and FD003 to 256 and 248 engines in FD002 and FD004, respectively. It is observed that the predicted RUL values closely follow their ground truths.

Three key points can be highlighted. First, it can be observed that the accuracy for engines with smaller RULs is noticeably higher, which is particularly important since a smaller RUL means a higher probability of a potential failure. Maintenance activities can be carried out in advance to avoid catastrophic failures. Second, it is easy to find that the prediction error is greater in the early stage than in the late stage, especially when the machine is in a fresh healthy state. That is, because each engine starts with different degrees of initial wear and manufacturing variations that are unknown, the prediction error is increased in the early stage. Third, the prediction accuracies shown in Figures 8(b) and 8(d) are poorer than the others because subsets FD002 and FD004 are the most complex scenarios, which make them more difficult to accurately predict the target RUL.

### 6.2. Comparison with the Literature

Studies that have reported results on all four subsets in the C-MAPSS dataset have been selected for comparison. Although the initial RUL values are somewhat different, the results are still comparable. As shown in Tables 5 and 6, the proposed deep architecture achieved promising results compared with the recent studies.

As seen from Table 5, the proposed deep architecture indicates a substantially improved RMSE prediction accuracy on all subsets over the other methods. This result means that the predicted RULs in the proposed architecture are closer to the real RULs. Due to the high reliability requirements of aircraft systems, a higher RUL prediction accuracy means more accurate and timely maintenance, which can greatly improve the safety of systems. In Table 6, the proposed approach achieves a better performance in the *S* metric for the FD001 and FD002 subsets. Although the remaining subsets did not reach the best performance, they were close to the best accuracy.

Compared with these algorithms, the performance of our model was first assessed through a parameter study only on subset FD001 to find suitable key parameters, and then it was directly applied to the other datasets without further tuning for each dataset. More importantly, the proposed scheme shows a good generalization capability for the other datasets when tuned only on subset FD001. Based on the comparison of the above two evaluation criteria, it can be seen that the proposed model greatly improves the prediction accuracy of the RUL of aircraft engines. However, for the two subsets of FD002 and FD004, the inherent complexity of the data increases the difficulty of extracting high-level abstract features, so the prediction stability remains to be improved.

## 7. Conclusions and Future Work

In this paper, we proposed and demonstrated a new deep learning approach, referred to as the DCGAN-based AE scheme, for RUL estimation from multivariate time-series sensor signals. The DCGAN and an AE are integrated to achieve a pretrained stage to extract high-level abstract representations from initial data, and then a fine-tuning stage that includes the LSTM and FNN is used to predict RUL. The improved results have proven that the pretraining model can capture the degradation trend of a fault, which means the proposed method can also be used as an efficient feature extraction scheme to solve other problems. Experiments are carried out on the popular C-MAPSS dataset to show the superiority of the proposed model. Comparisons with several state-of-the-art approaches demonstrate better prediction performance of the model, which proves that the proposed data-driven prognostic method is effective and suitable for prediction problems.

While good experimental results were obtained by the proposed method, further optimization is still necessary. Improving the stability of the method for complex conditions is a further direction for future research. Moreover, efforts should be made to decrease the average training time for each subset in the future.

## Figures and Tables

**Algorithm 1:**
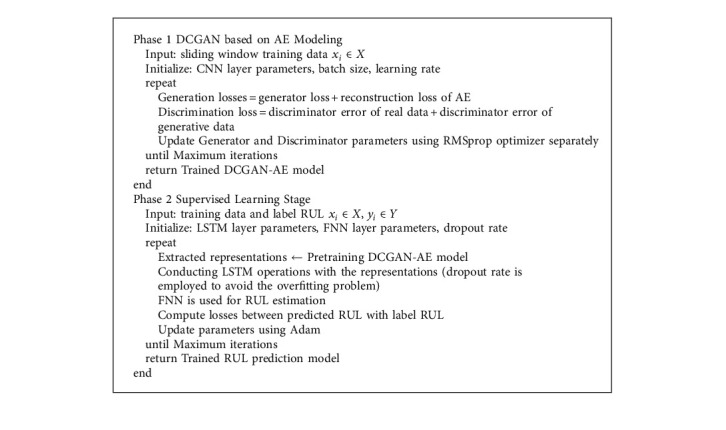
Outline of proposed model training for RUL estimation.

**Table 1:** Recent deep learning (DL) approaches proposed for RUL predictions on the C-MAPSS dataset (the years between 2016 and 2019).

Authors and references	Year	Approach
Babu et al. [23]	2016	CNN + FNN
Zhang et al. [28]	2016	MODBNE
Zheng et al. [20]	2017	LSTM + FNN
Li et al. [8]	2018	CNN + FNN
Yu et al. [27]	2019	BiLSTM-ED
Ellefsen et al. [24]	2019	RBM + LSTM

**Table 2:** Variables of the C-MAPSS dataset.

Sensor data number	Description	Units
1	Total temperature at the fan inlet	°R
2	Total temperature at the low-pressure compressor outlet	°R
3	Total temperature at the high-pressure compressor outlet	°R
4	Total temperature at the low-pressure turbine outlet	°R
5	Pressure at the fan inlet	psia
6	Total pressure in bypass-duct	psia
7	Total pressure at the high-pressure compressor outlet	psia
8	Physical fan speed	rpm
9	Physical core speed	rpm
10	Engine pressure ratio	—
11	Static pressure at the high-pressure compressor outlet (Ps30)	psia
12	Ratio of fuel flow to Ps30	pps/psi
13	Corrected fan speed	rpm
14	Corrected core speed	rpm
15	Bypass ratio	—
16	Burner fuel-air ratio	—
17	Bleed enthalpy	—
18	Demanded fan speed	rpm
19	Demanded corrected fan speed	rpm
20	High-pressure turbine coolant bleed	lbm/s
21	Low-pressure turbine coolant bleed	lbm/s

**Table 3:** Details of the C-MAPSS dataset.

Dataset	FD001	FD002	FD003	FD004
Engines of the training set	100	260	100	249
Engines of the test set	100	259	100	248
Fault modes	1	1	2	2
Operational modes	1	6	1	6
Training samples (default)	17,731	48,819	21,820	57,522
Testing samples	100	259	100	248

**Table 4:** Default parameters of the supervised architecture.

Architecture	Hidden size	Dropout	Activation function
First LSTM layer	64	0.5	tan*h*
Second LSTM layer	64	0.5	tan*h*
First FNN layer	32	0.5	ReLU
Second FNN layer	1	1.0	Abs

**Table 5:** RMSE comparison with the literature on the C-MAPSS dataset.

DL approach and references	FD001	FD002	FD003	FD004
CNN + FNN [23]	18.45	30.29	19.82	29.16
MODBNE [28]	15.04	25.05	12.51	28.66
LSTM + FNN [20]	16.14	24.49	16.18	28.17
CNN + FNN [8]	12.61	22.36	12.64	23.31
BiLSTM-ED [27]	14.74	22.07	17.48	23.49
RBM + LSTM [24]	12.56	22.73	12.10	22.66
Proposed architecture	10.71	19.49	11.48	19.71

**Table 6:** Score function comparison with the literature on the C-MAPSS dataset.

DL approach and references	FD001	FD002	FD003	FD004
CNN + FNN [23]	1287	13,570	1596	7886
MODBNE [28]	334	5585	422	6558
LSTM + FNN [20]	338	4450	852	5550
CNN + FNN [8]	274	10,412	284	12,466
BiLSTM-ED [27]	273	3099	574	3202
RBM + LSTM [24]	231	3366	251	2840
Proposed architecture	174	2982	273	3874

## Data Availability

The dataset was provided by the Prognostics CoE at NASA Ames. So, this dataset is public, and we can visit https://ti.arc.nasa.gov/tech/dash/groups/pcoe/prognostic-data-repository/ to get it. The dataset we use is the sixth in all datasets from the website, and the name is “Turbofan Engine Degradation Simulation Data Set.” The dataset is in the text format and has been zipped including a readme file.
